# Indications and outcome of repeat penetrating keratoplasty in India

**DOI:** 10.1186/1471-2415-5-26

**Published:** 2005-11-02

**Authors:** M Vanathi, Namrata Sharma, Rajesh Sinha, Radhika Tandon, Jeewan S Titiyal, Rasik B Vajpayee

**Affiliations:** 1 Rajendra Prasad Centre For Ophthalmic Sciences, All India Institute of Medical Sciences, New Delhi, India

## Abstract

**Background:**

Repeat penetrating keratoplasty is quite often required as there is high chance of failure of the primary graft particularly in the developing world. We planned a study to analyze the indications and outcome of repeat penetrating keratoplasty in a tertiary care centre in India.

**Methods:**

A retrospective analysis of all the patients who underwent repeat penetrating keratoplasty, between January 1999 and December 2001 was performed. The parameters evaluated were indication for the primary penetrating keratoplasty, causes of failure of the previous graft, and final visual outcome and clarity of the repeat corneal grafts.

**Results:**

Of fifty-three eyes of 50 patients with repeat penetrating keratoplasty (three patients underwent bilateral corneal regrafts), 37 eyes had undergone one regraft each, 14 eyes two regrafts and two eyes had three regrafts. The follow-up of the patients ranged from one to three years. The most common primary etiologic diagnosis was vascularized corneal scars (66%), of which the scars related to infection were most common (68.5%). Twenty-eight regrafts (52.8%) remained clear at a mean follow-up of 1.54 ± 0.68 years, of which 25 were single regrafts (89.3%). The commonest cause of failure of regraft was infection to the corneal graft (recurrence of herpetic infection in 9 eyes and perforated graft ulcers in 3 eyes). Three (18.6%) of the 16 eyes with multiple corneal regrafts achieved a BCVA of 6/60. Overall, only five eyes (all with single regraft) achieved a BCVA of 6/18 or better at the end of follow-up.

**Conclusion:**

Graft infection is the leading cause of failure of repeat keratoplasty in this part of the world. Prognosis for visual recovery and graft survival is worse in eyes undergoing multiple regrafts.

## Background

Corneal graft failure constitutes a common indication for penetrating keratoplasty [1-20]. There are a few studies in literature which have reported the indications and outcome of corneal regrafts [21-27]. The primary indications for repeat corneal transplantation show a changing trend. Aphakic and pseudophakic bullous keratopathy were the common primary indications for regrafts in developed countries in previous studies [22-26], others being, infectious keratitis [23] and corneal dystrophies [22,25]. However, a more recent study on the profile of repeat keratoplasty [27] identified vascularized corneal scar as the most common primary indication for corneal regrafting. The paucity of studies on repeat keratoplasty from developing countries prompted us to evaluate the indications and outcome of repeat keratoplasty at a tertiary eye care referral centre.

## Methods

We retrospectively reviewed the records of 50 patients of corneal regrafts performed at the Cornea services of Rajendra Prasad Centre for Ophthalmic Sciences, All India Institute of Medical Sciences, New Delhi, during the period from January 1999 to December 2001. Of all these 50 patients, 32 patients (64%) were from rural and peri-urban areas and 18 (36%) were from urban areas.

The parameters evaluated were the patient's age, gender, indication for the primary corneal transplan tation, duration of follow-up, number of regrafts, associated procedures performed and outcome of corneal regrafts. More than one corneal regraft was considered as a multiple regraft. Details of previous grafts in cases of multiple regrafts were collected from old records wherever available. Graft outcome was defined in terms of the clarity over a period of time till last follow-up or graft failure, whichever was earlier. Graft was considered to be clear if the clarity was of grade 3 or grade 4. By grade 4 clarity, we mean an absolutely clear graft with good visualization of the iris details behind it. By grade 3 clarity, we mean a graft with minimal haze but still with reasonably good visualization of the iris details.

Allograft rejection was diagnosed by the presence of endothelial or epithelial rejection line or both and corneal edema with anterior chamber reaction. Corneal graft failure was diagnosed when irreversible graft edema was present with or without vascularization or scarring of the graft. Intraocular pressure greater than 21 mm Hg on two separate occasions was taken as secondary glaucoma.

Postoperatively patients were prescribed topical betamethasone sodium phosphate 0.1% and ciprofloxacin 0.3% QID each and ocular lubricants (polyvinyl alcohol) QID. Patients with healed herpetic keratitis were given oral acyclovir 400 mg twice a day for 1 year after keratoplasty. The follow up schedule after the surgery was daily from day 1 till epithelial healing, 1 week, 1 month, 3 months, 6 months, 1 year and yearly thereafter. The duration of follow-up was taken as the time between the last regraft and the final follow-up. The outcome of the surgery was analyzed statistically using paired 't' test and p-value smaller than 0.05 was considered as statistically significant.

## Results

Sixty eight patients had undergone corneal regrafts at our centre during the study period. Of these, 50 patients (73.5 %) (41 males and 9 females) had a regular follow-up with us of which, 3 patients had undergone bilateral corneal regrafts and these were included in the study (N = 53 eyes). The primary keratoplasty was performed at our centre in 39 eyes of 36 patients and 14 patients were referred from other centres after failure of the primary graft.

The mean age of the patients at the time of repeat penetrating keratoplasty was 45.2 ± 16.5 years. The mean follow-up after regraft was 1.54 ± 0.68 years. Of a total of 53 eyes, 37 eyes had one corneal regraft, 14 had two corneal regrafts and two eyes had undergone three regrafts each (i.e. multiple regrafts in 16 eyes). Forty-eight eyes had undergone other associated intraocular procedures such as goniosynechiolysis (32 eyes), iridectomy with pupilloplasty (7 eyes), cataract extraction (7 eyes) and anterior vitrectomy (11 eyes) at the time of regraft.

The most common indication for primary penetrating keratoplasty in these eyes was vascularized corneal scars (35 of the 53 eyes; 66%) followed by perforations secondary to microbial keratitis (6/ 53; 11.3%) (Table 1). The most common cause of vascularized corneal scars was healed microbial keratitis (non-herpetic) (37.1%), followed by herpetic keratitis (31.4%) (Table 1). The amount of vascularization was variable. Fifteen eyes showed a single quadrant deep vascularization along with one quadrant of superficial vascularization; eleven eyes showed one quadrant deep vascularization and 2 quadrants of superficial vascularization; 8 eyes had 2 quadrants of deep vascularization and 2 quadrants of superficial vascularization, and 1 eye had 3 quadrants of deep and 3 quadrants of superficial corneal vascularization.

The failure of primary graft was attributable to poor ocular surface in 18 eyes (33.9%), recurrence of herpetic keratitis in 8 eyes (15%), perforated graft ulcers in 4 (7.5%), scarring due to graft infection in 4 (7.5%), allograft rejection in 7 eyes (13.2%), endothelial decompensation in 8 eyes (15 %) and raised intraocular pressure in 4 eyes (7.5%). Since a high proportion of the grafts failed due to poor ocular surface, all these eyes were put on intensive (1 hourly) preservative free lubricants and repeat grafting was performed only after these eyes attained a reasonably good ocular surface. Two eyes with failed primary graft and ocular surface problems required entropion surgery and in two eyes permanent punctual plugs were inserted before regraft.

After repeat graft, these eyes were prescribed 1 hourly preservative free lubricant for 3 months and QID later along with topical chloramphenicol and topical dexamethasone QID each for 3 months and BD each later.

**Figure 1:**
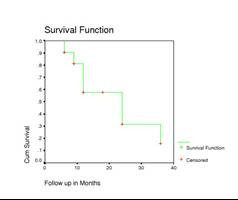
Corneal graft survival.

Of the 53 eyes with regrafts, 28 eyes (52.8%) had clear grafts at the end of follow up of which, 25 eyes (89.3%) had undergone single regraft and 3 eyes had multiple regrafts (Table 2). The reasons for failure in the remaining 25 eyes (47.2 %) was recurrence of herpetic infection in 9 eyes (36%), uncontrolled glaucoma in 5 eyes (20%), allograft rejection in 4 eyes (16%), perforated graft ulcers in 3 eyes (12%), endothelial decompensation in 2 eyes (8%), poor ocular surface in 2 eyes (8%). The survival of the regrafts has been depicted in Figure 1.

The pre-operative visual acuity ranged from light perception to 1/60. The best corrected visual acuity of the 28 clear grafts ranged from 4/60 to 6/9. Only five eyes (9.4%) achieved a BCVA of 6/18 or better at the end of 1 year after re graft, of which all had single regrafts (Table 3). In twenty-five eyes with failed repeat grafts, visual acuity ranged from light perception to 1/60.

Causes of suboptimal visual outcome (post-refraction) in regrafts that remained clear (grade 3/4) was poor ocular surface (9 eyes), post penetrating keratoplasty astigmatism (6 eyes), macular scarring (5 eyes), treated graft rejection (4 eyes) and post-penetrating keratoplasty glaucoma (4 eyes).

## Discussion

Repeat corneal grafts remain a drain on existing resources for corneal transplantation and the rise in the number of regrafts parallels the rise in number of primary keratoplasties. Several studies [1-20] on the indications for penetrating keratoplasty have cited variable figures comprising the proportion of regrafts varying from 6.6% to 41%. On the basis of these studies it is apparent that corneal regrafts show an increasing trend among indications for penetrating keratoplasty. Dandona et al [19] studied the indications for penetrating keratoplasty in India and reported regrafts as the second most common indication for penetrating keratoplasty in India (17.1%), next only to corneal scarring of varied etiology.

Different success rates and visual outcome in corneal regrafts have been reported in literature [21-27]. Reported percentages of clear regrafts vary from 51% to 74% in the earlier studies [21,25-27]. In the present study the outcome of repeat penetrating keratoplasty in terms of graft clarity was comparable (52.8%).

Eyes in which repeat keratoplasty was performed necessitated multiple intraocular manipulations like goniosynechiolysis, iridectomy with pupilloplasty, cataract extraction and anterior vitrectomy. Post-keratoplasty glaucoma resulting due to these manipulations is an important factor that can result in graft failure. In the present study, a higher proportion of eyes developed secondary glaucoma (5 out of 25) resulting in graft failure in regrafts in comparison to primary keratoplasty (4 out of 53). This perhaps can be attributed to the fact that repeated surgeries result in greater development of anterior chamber reaction and multiple synechiae, and multiple intraocular manipulations required in regrafts can further be the compounding factor for the same.

Patients who undergo repeat corneal grafts carry the risk of developing variable amount of corneal neovascularisation as was seen in our cases. Corneal neovascularisation is an independent risk factor that can jeopardize the outcome of a successfully performed keratoplasty by causing episodes of graft rejection. However, in contrast to the studies from the developed countries which report graft rejection and recurrence of dystrophies as the main causes for failure of regrafts [22-25], our study highlights that higher prevalence of graft infection including recurrence of previous infection and secondary glaucoma are the leading causes of failure of repeat grafts. Ocular surface problem was the leading cause of failure of primary graft in this study. These eyes were intensively treated with preservative free lubricants to improve the ocular surface before performing regraft. This improved graft survival in these eyes and very few regrafts failed due to ocular surface problems. However, some amount of ocular surface problem persisted in some eyes resulting in mild haze and hence remained the leading cause for suboptimal best corrected visual acuity in these regrafts in spite of reasonably good graft clarity.

Recent studies [25-27] report a visual acuity of 20/40 or better in only 15% to 41% of clear regrafts. Similarly, the present study reports that among the 28 eyes with clear regrafts, a BCVA of 6/18 or better was seen in five eyes only (17.9%) and less than 6/18 in 23 eyes (82.1%). Contrary to few studies [22,25,26] reporting that visual prognosis in multiple regrafts to be comparable to that in single regrafts (these regrafts had been performed mainly for pseudophakic bullous keratopathy), Bersudsky et al [27] had concluded that graft survival and visual outcome is inversely proportional to the number of corneal regrafts. Similarly our study also demonstrates a poorer visual outcome in eyes undergoing multiple regrafts (Table 3). Though the number of eyes with multiple regrafts was small in our study, 13 eyes (81.3%) out of 16 eyes with multiple regrafts failed in our study suggesting that prognosis for graft survival in multiple regrafts seems to be poorer. Owing to the poor graft survival, permanent keratoprosthesis may be a suitable alternative in eyes with multiple regrafts.

## Conclusion

The study demonstrates that visual prognosis for multiple corneal regrafting is suboptimal, owing to high chance of graft infection, secondary glaucoma and allograft rejection. However, repeat penetrating keratoplasty should be considered when required depending upon patient's need and motivation and in the absence of any contraindication.

## Competing interests

The author(s) declare that they have no competing interests.

## Authors' contributions

MV performed data collection, NS designed the study, RS wrote the manuscript, RT followed up and evaluated the patients, JST and RBV performed the surgeries. All authors read and approved the manuscript.

## Pre-publication history

The pre-publication history for this paper can be accessed here:



## Figures and Tables

**Table 1:** Indications for primary corneal transplantation in corneal regrafts

	Indications	No of eyes (%) (n = 53)	Single regraft (n = 37)	Multiple Regraft (n = 16)
1	Vascularised corneal scars	35 (66)	22	13
	Healed infectious keratitis (non-herpetic)	13 (37.1)	9	4
	Herpetic scarring	11 (31.4)	3	8
	Trachomatous	5 (14.3)	5	0
	Trauma	4 (11.4)	4	0
	Steven Johnson's syndrome	1 (2.9)	1	0
	Aniridia	1 (2.9)	0	1
2	Infectious keratitis	6 (11.3)	5	1
3	Aphakic Bullous Keratopathy	4 (7.5)	3	1
4	Pseudophakic Bullous Keratopathy	3 (5.7)	3	0
5	Corneal dystrophy	4 (7.5)	3	1
6	Congenital glaucoma	1 (1.9)	1	0

**Table 2:** Outcome of corneal regrafting surgery

		Multiple regrafts	
No of eyes (n = 53)	1 regraft (n = 37)	2 regraft (n = 14)	3 regraft (n = 2)
Clear regrafts (n = 28)	25	3	0
Failed regrafts (n = 25)	12	11	2
p value (Paired 't' test)	0.001 (statistically significant)	0.006 (not significant)	

**Table 3:** Visual Acuity of single Vs multiple regrafts at last follow up

Clear regrafts (n = 28)	BCVA 6/18 or better (n = 5)	BCVA Less than 6/18 (n = 23)
Single regrafts (n = 25)	5	20
Multiple regrafts (n = 3)	0	3
